# Optimized protocol for isolation of neutrophils from small-volume human blood samples via Percoll density gradient centrifugation

**DOI:** 10.1016/j.xpro.2026.104914

**Published:** 2026-10-28

**Authors:** Rui She, Shaotian Chen, Zhiquan Liu, Xuemei Tang, Xiang Qiu, Weijian Chen

**Affiliations:** 1 Department of Rheumatology & Immunology, Children’s Hospital of Chongqing Medical University, National Clinical Research Center for Children and Adolescents’ Health and Diseases, Ministry of Education Key Laboratory of Child Development and Disorders, Chongqing Key Laboratory of Child Rare Diseases in Infection and Immunity, Chongqing 400014, China

**Keywords:** Cell isolation, Health Sciences, Immunology

## Abstract

Neutrophils are critical first-line innate immune defenders. Conventional Percoll-based isolation protocols perform reliably only with large blood volumes (e.g., 10 mL) and yield inconsistent results from small-volume samples (≤2 mL), such as those from pediatric individuals. Here, we present an optimized protocol tailored for minimal-volume specimens, with systematic adjustments to centrifugation speed, duration, and gradient setup to ensure neutrophil isolation with high purity and viability. Among healthy donor samples (*n* = 19), the protocol yielded 1.74 ± 0.57 × 10^6^ granulocytes per mL of blood. Across all tested samples (*n* = 22), granulocyte purity was 91.58% ± 3.70%, and neutrophils comprised 96.48% ± 1.75% of recovered granulocytes in six evaluated isolates. In comparison, conventional protocols yielded granulocytes with low purity of 33.25% ± 13.99%. This robust, reproducible method enables reliable neutrophil isolation, expanding the accessibility of neutrophil-related research to scenarios with limited blood volume.

## Highlights

• Optimized neutrophil isolation protocol for small-volume pediatric peripheral blood
• High granulocyte recovery with high neutrophil purity and viability
• Tuned Percoll gradient, adjusted spins, and neutrophil purity validation

## Graphical abstract

**Figure:**
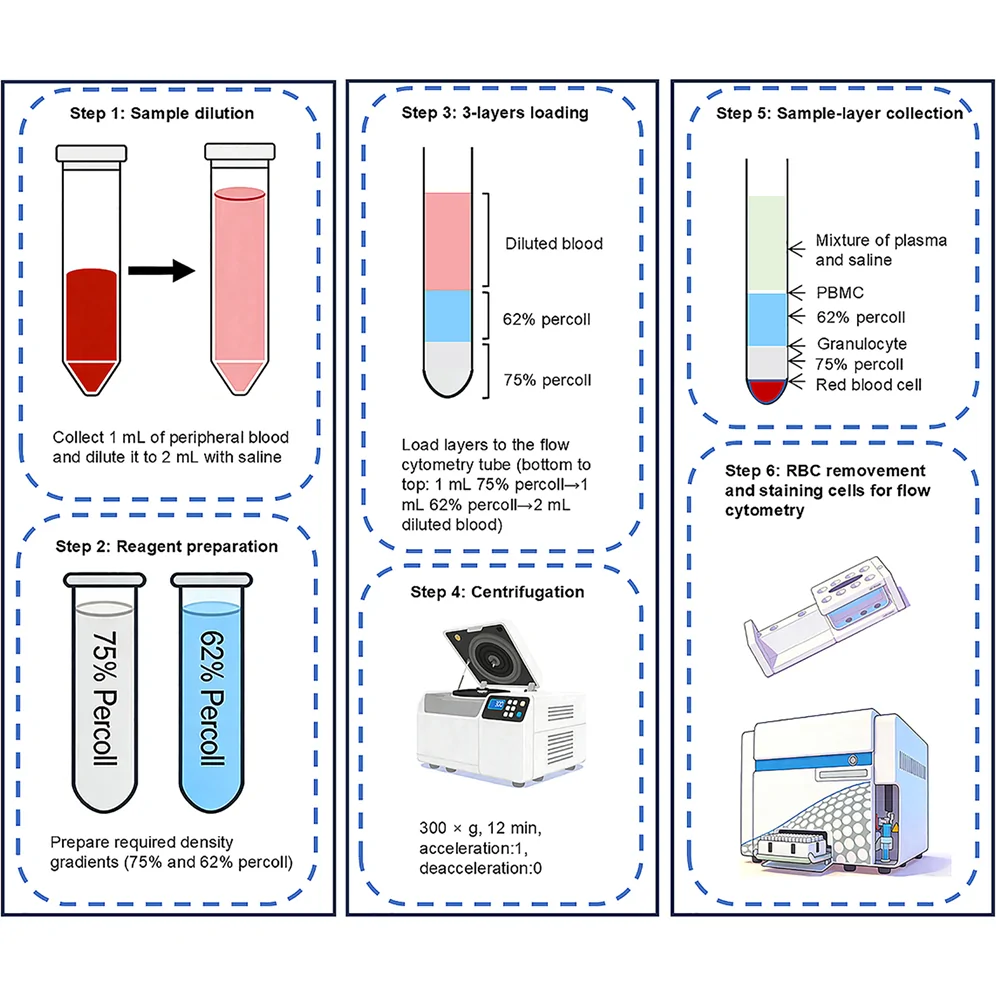

## Teaser

Limited blood availability complicates neutrophil isolation in pediatric research. She et al. describe an optimized Percoll density gradient protocol for isolating viable neutrophils from 1 mL of peripheral blood. Optimized centrifugation conditions improve granulocyte recovery and purity, enabling neutrophil studies with small blood volumes.

## Introduction

Neutrophils are the most abundant leukocytes in human peripheral blood and play a pivotal role in innate immunity, inflammation, and host defense against pathogens.^1^ They are frequently investigated in the context of infectious diseases, sepsis, autoimmune disorders, and cancer, serving as a key research focus in clinical and basic immunology.

Conventional density gradient centrifugation protocols using Percoll are well-established for isolating neutrophils,^2^ but this method is typically optimized for large-volume peripheral blood samples. In clinical practice, however, the volume of peripheral blood obtainable from patients (especially pediatric, neonate, or critically ill individuals) is often limited, making it challenging to apply conventional isolation protocols. Specifically, when processing small-volume blood samples, the conventional protocols frequently result in low isolation efficiency, or insufficient purity—limiting their clinical applicability and research utility.

To address this limitation, we refined the Percoll density gradient centrifugation protocol to accommodate small-volume peripheral blood samples for neutrophil isolation, with validation in terms of yield and purity. Collectively, this protocol offers distinct advantages: 1) Isolate neutrophils based on small-volume samples for research (e.g., children with limited availability), reserving individual sample specificity; 2) Low cost and possibly causes less damage to cell viability^3^^,^^4^ compared to magnetic bead sorting or flow cytometry sorting; 3) Facilitates research on neutrophils^5^^,^^6^—key participants in inflammatory responses—by filling the gap in small-volume neutrophils isolation, thereby expanding the accessibility of neutrophil-related research to scenarios with limited blood volume.

## Results and discussion

### Limitations of conventional Percoll protocols for minimal-volume peripheral blood samples

Conventional Percoll density gradient protocols are well-established for isolating neutrophils from large-volume human peripheral blood (e.g., 10 mL). However, when these conventional protocols were directly applied to minimal-volume samples (e.g., 1 mL), they failed to achieve effective neutrophil isolation (Figure 1). Specifically, the purity of the recovered granulocyte fraction was markedly reduced (Figure 1; Table S1), such that the proportion of neutrophils in the isolated fraction could not even be reliably determined, rendering the preparation inadequate for downstream neutrophil-related research. These results clearly indicate that conventional protocols are not suitable for minimal-volume blood samples, possibly due to excessive cell loss caused by improper centrifugation parameters during small-volume processing.

**Figure 1:**
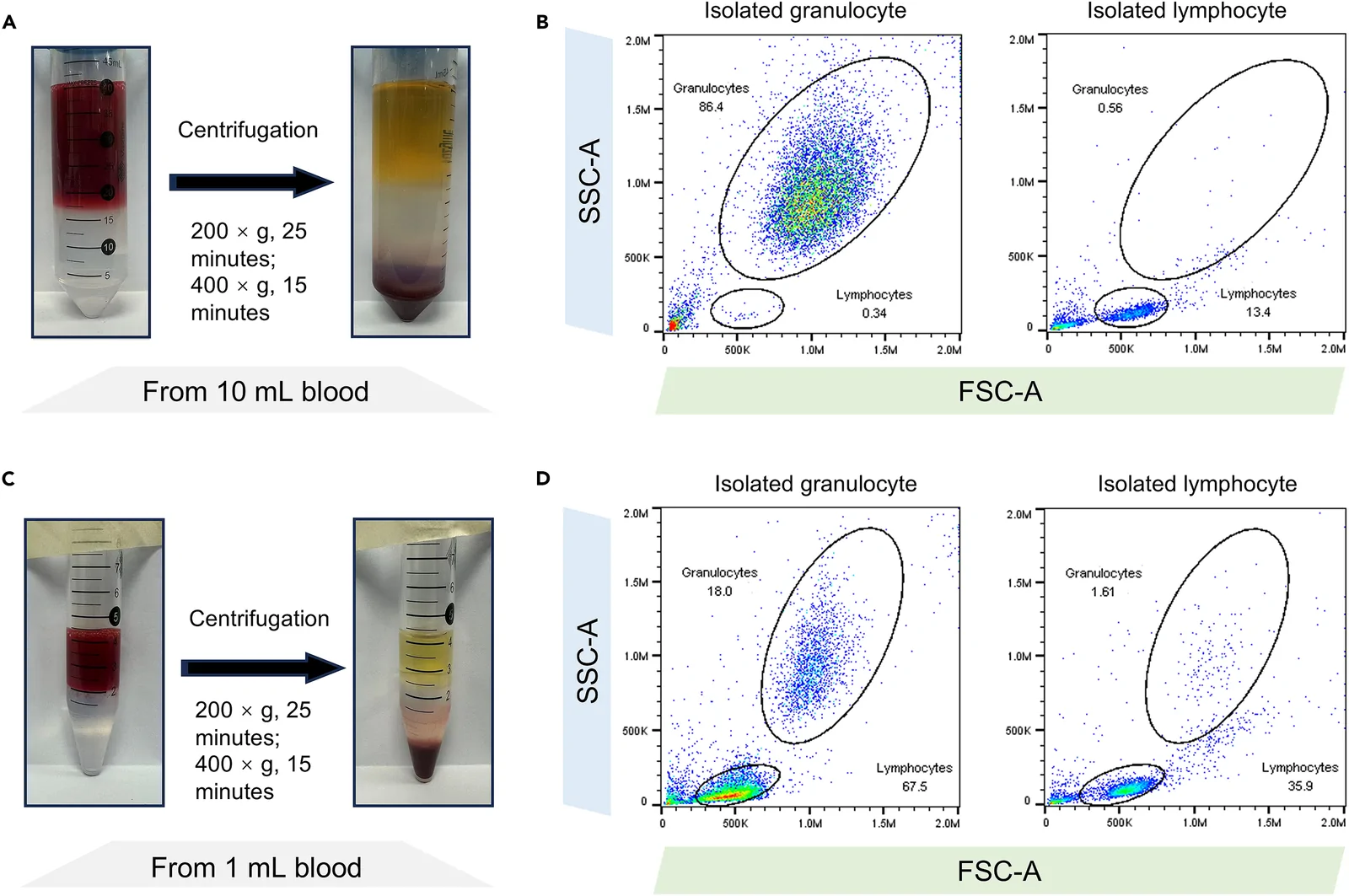
Neutrophil isolation from varied human blood volumes using Percoll density gradient centrifugation (A) Pictures of double layer Percoll gradient before (left) and after (right) centrifugation, using large-volume human blood. (B) After Percoll gradient, granulocytes (mainly neutrophils) found between the Percoll layers are successfully isolated and high purity was shown in flow plots. (C) Pictures of double layer Percoll gradient before (left) and after (right) centrifugation, using small-volume human blood. (D) After Percoll gradient, Granulocytes (mainly neutrophils) found between the Percoll layers are isolated with substantial lymphocyte contamination and lower granulocyte purity and lower purity was shown in flow plots.

### Optimization of key parameters for minimal-volume neutrophil isolation

To address the limitations of conventional methods, we refined several key parameters of the Percoll density gradient protocol, including the blood-to-saline dilution ratio, Percoll gradient concentration, centrifugation speed, and centrifugation time. After optimization, the protocol was successfully adapted to 1 mL of human peripheral blood samples (Figures 2A–2D), and was also successfully applied to representative samples from patients with Kawasaki disease (Figures 2E and 2F; Table S2). Furthermore, among healthy-donor samples (n=19), the granulocyte yield was 1.74 ± 0.57 ×10^6^ cells per mL of blood. Across all tested samples (19 healthy donors and 3 patients with KD; n=22), granulocyte purity was 91.58 ± 3.70% (Table S2). Cell viability was assessed in four independent isolates, yielding values of 98.33%, 97.75%, 96.52% and 97.19%, supporting robust cell integrity for downstream applications. Most importantly, this optimized protocol effectively addressed the problem of low granulocyte recovery from small-volume samples, enabling reliable isolation of neutrophils from minimal-volume peripheral blood that was previously difficult to achieve with conventional method.

**Figure 2:**
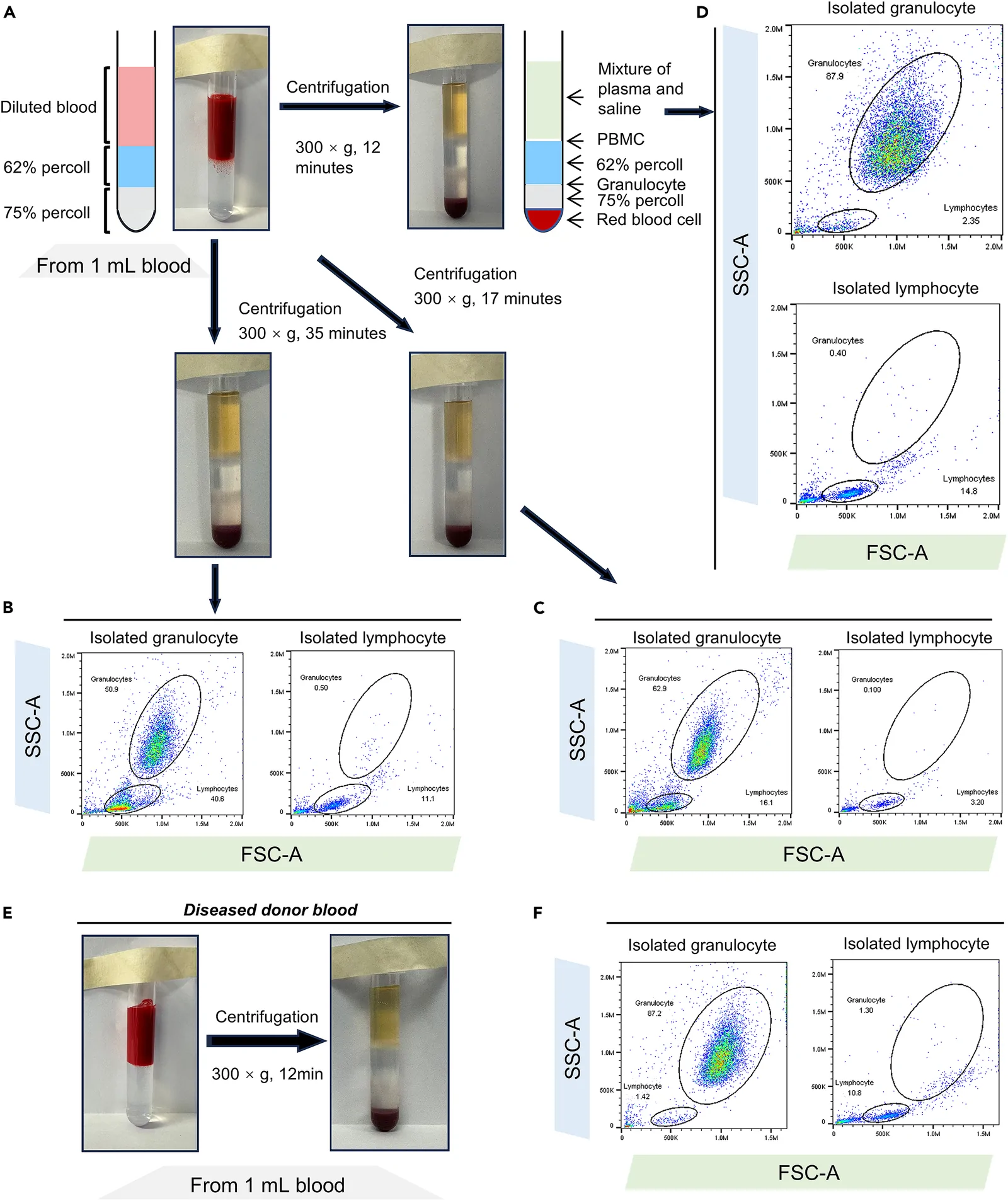
Refined centrifugation setting for neutrophil isolation from small-volume human blood (A) Draw and pictures of double layer Percoll gradient before (left) and after (right) centrifugation, using small-volume healthy donor blood, with different centrifugation setting. (B, C and D) After Percoll gradient with indicated centrifugation time, granulocytes (mainly neutrophils) found between the Percoll layers are isolated and purity of each was shown, among which centrifugation at 300 × g for 12 min yielded the best separation performance among the tested conditions. (E) Pictures of double layer Percoll gradient before (left) and after (right) centrifugation, using small-volume diseased donor blood. (F) After Percoll gradient, granulocytes (mainly neutrophils) found between the Percoll layers are isolated and exhibit high purity.

### High purity of neutrophils isolated by the optimized protocol

To verify the specificity and reliability of the optimized protocol, we assessed the purity of neutrophils among the isolated granulocytes using flow cytometry (defined as CD45^+^ 7-AAD^-^CD66b^+^ cells) in six isolates. With the yielded values of 95.3%, 94.3%, 96.6%, 96.1%, 97.2%, and 99.4%, these results showed that neutrophils accounted for 96.48% ± 1.75% of the isolated granulocytes (Figure 3; Table S3), confirming that the optimized protocol not only improves the yield of granulocytes from minimal-volume samples but also ensures high purity of the target neutrophils. This high purity indicates that the optimized protocol effectively provides high-quality neutrophils for subsequent functional or molecular experiments.

**Figure 3:**
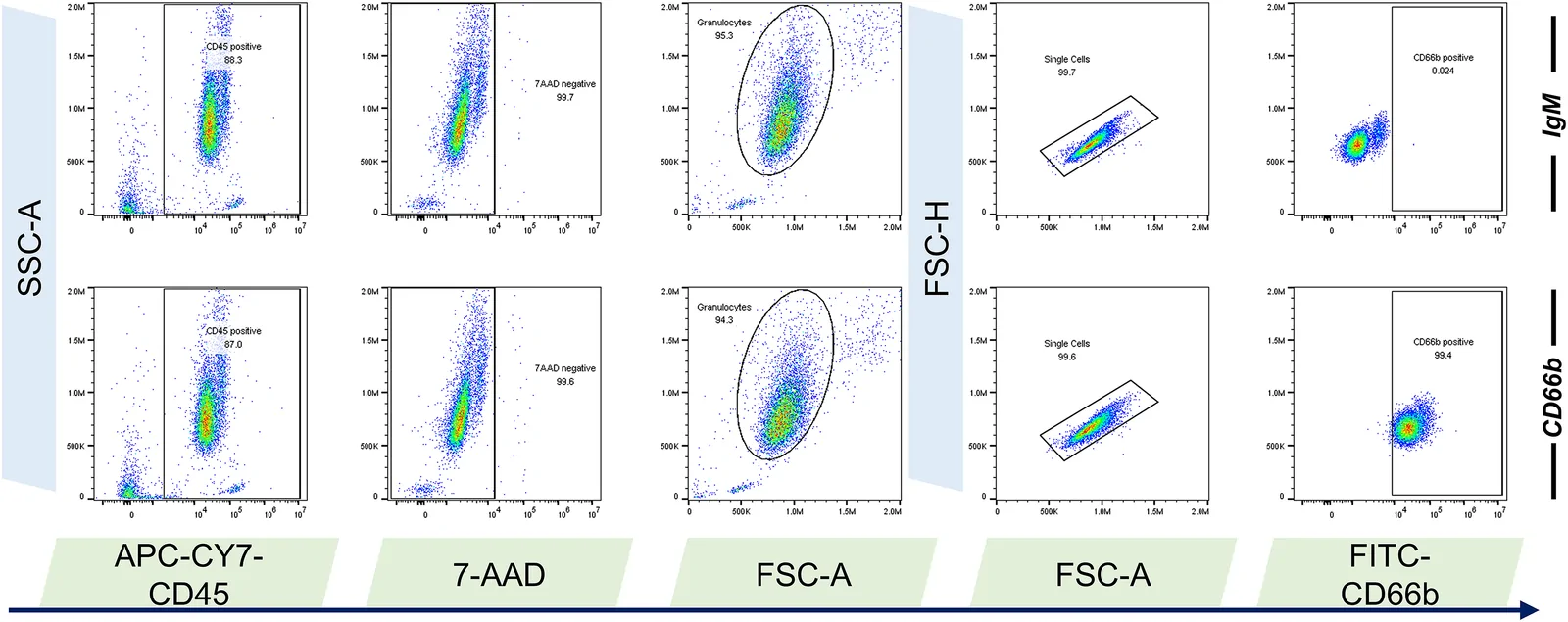
Gating strategy for mature neutrophils Neutrophils were identified as CD66b^+^ cells within viable singlet CD45^+^ granulocytes. Flow plots from left to right illustrate the percentage of neutrophils within singlet viable granulocytes gated from total leukocytes and high purity of isolated neutrophils was proved.

## Discussion

This protocol achieves reliable isolation of neutrophils from small-volume pediatric peripheral blood samples, addressing a critical unmet need in clinical and research settings. The use of a Percoll density gradient for neutrophil isolation offers significant advantages over bead-based and FACS-based methods^3^^,^^4^: it yields a highly pure cell population (neutrophils account for 96.48% ± 1.75% of the isolated granulocytes), which is defined as CD45^+^ 7-AAD^-^ CD66b^+^ with minimal contamination and high viability.

In this study, we optimized the vessel type and centrifugation parameters to establish a robust protocol for high-purity neutrophil isolation from small-volume peripheral blood samples. Compared with conventional 15 mL centrifuge tubes, FACS tubes offer distinct advantages for the processing of microscale blood specimens. Owing to the inherently limited granulocyte count in small-volume blood samples, density gradient centrifugation yields only an extremely thin granulocyte layer, which is structurally unstable and difficult to visualize in large-caliber centrifuge tubes. By contrast, the reduced cross-sectional diameter of FACS tubes concentrates cell populations within a narrower vertical plane, facilitating stable formation of thin granulocyte layers and preventing excessive dispersion of target cells. Additionally, the fully transparent wall of FACS tubes allows direct visual monitoring of stratified interfaces, enabling intuitive evaluation of layering integrity and timely detection of aberrant separation.

Importantly, the use of FACS tubes is highly compatible with our optimized mild centrifugation condition (300 × g for 12 minutes). The original higher-force, prolonged centrifugation parameters designed for large-volume blood samples are overly stringent for microscale specimens, as they induce non-specific sedimentation of peripheral blood mononuclear cells (PBMCs) and consequently contaminate the granulocyte layer. Combining the structural superiority of FACS tubes with milder centrifugation settings, our refined protocol effectively reduces PBMC contamination and achieves consistent, reliable stratification of small-volume blood samples. Of note, this optimized strategy yields high-purity neutrophils across the healthy and Kawasaki disease (KD) samples tested in this study, substantially improving the general applicability and technical robustness of neutrophil isolation from small-volume blood.

This protocol addresses the technical gap in efficient neutrophil purification from limited blood volumes, making it particularly suitable for clinical scenarios where large-volume blood collection is impractical, such as pediatric patients and critically ill individuals with restricted blood accessibility. Furthermore, this simple and cost-effective method serves as a feasible alternative to sophisticated flow sorting-based neutrophil isolation approaches, greatly improving its accessibility for routine basic and translational research. Collectively, this optimized protocol provides a dependable and reproducible strategy for harvesting high-quality primary neutrophils, which supports future investigations into neutrophil biology and related clinical translational studies.

### Limitations

In this study, we focus on isolating neutrophils from pediatric peripheral blood samples. Although this method yields a neutrophil population with high purity (defined as CD45^+^ 7-AAD^-^CD66b^+^), careful handling is required to minimize contamination. Furthermore, it is recommended to use fresh blood as the source for neutrophil isolation.

## Resource availability

### Lead contact

Further information and requests for resources and reagents should be directed to and will be fulfilled by the lead contact, Weijian Chen (weijianchen@hospital.cqmu.edu.cn).

### Technical contact

Technical inquiries, experimental troubleshooting, and protocol operation guidance should be directed to and will be fulfilled by the technical contact, Weijian Chen (weijianchen@hospital.cqmu.edu.cn).

### Materials availability

This study did not generate new unique reagents.

### Data and code availability

All data generated or analyzed in this study are included in the published article and its supplemental information. Raw flow cytometry data and additional information required to reanalyze the data are available from the lead contact upon reasonable request.

## Acknowledgments

This study was supported by the 10.13039/100014718National Natural Science Foundation of China (no. 82571780 to X.Q.). We sincerely apologize to any authors whose work we may have inadvertently omitted in our reference list. All components of the graphical abstract were self-developed by the authors. The small icons in the figure were generated by converting original experimental photographs into cartoon-style images with Doubao, and no materials or templates from BioRender were adopted.

## Author contributions

R.S., S.C., X.T., X.Q., and W.C. conceived and designed the research. R.S., S.C., Z.L., and W.C. performed the experiments. R.S., S.C., X.T., X.Q., and W.C. interpreted the data and results and wrote the manuscript.

## Declaration of interests

The authors declare no competing interests.

## Declaration of generative AI and AI-assisted technologies in the manuscript preparation process

During the preparation of this work, we used Doubao and DeepSeek v.3.2 for language polishing. We reviewed and edited the output as needed and take full responsibility for the content of the published article.

## STAR★Methods

### Key resources table

**Table:** 

REAGENT or RESOURCES	SOURCE	IDENTIFIER
**Antibodies**		

FITC anti-human CD66b Antibody, Clone: G10F5, used as 1:20 dilution	Biolegend	305103
FITC Mouse IgM, κ Isotype Ctrl Antibody, Clone: MM-30, used as 1:20 dilution	Biolegend	401605
APC-Cy7 anti-human CD45, Clone:H130, used as 1:20 dilution	Biolegend	304014

**Biological samples**		

Human whole blood	N/A	N/A

**Chemicals, peptides, and recombinant proteins**		

Pure Percoll	Cytiva	GE17-0891-02
Sodium Chloride	BioFroxx	1249KG001
Saline	Solarbio	IN9000
PBS (Tablets)	Solarbio	P1000
FBS (Fetal Bovine Serum)	Thermo Fisher	A5256701
Trypan Blue Staining Solution (0.4%)	Biosharp	BL627A
7-AAD, used as 1:20 dilution	BD Pharmingen™	559925

**Software and algorithms**		

FlowJo V10.6.2	BD Biosciences	https://www.flowjo.com

**Other**		

1.5 mL Microtube	CORNING, Axygen	MCT-150-C
2 mL Microtube	CORNING, Axygen	MCT-200-C
15 mL Centrifuge tube	Vazyme	TCF0015A-02B/02C
50 mL Centrifuge tube	Vazyme	TCF0050A-02B/02C
5 mL Flow cytometry tube (FACS tube)	CORNING, Falcon	352054
EasySep™ RBC Depletion Reagent	STEMCELL Technologies	NC1620233
Modular magnetic rack (for 1.5 mL and 15 mL tubes)	TIANGEN	OSE-MF-01
EasySep™ Cell Separation Magnets	STEMCELL	18000
Hemocytometer	Countstar	C0010101/C0010101
Automatic Cell Analyzer	Countstar	Countstar Mira BF
High-speed refrigerated benchtop centrifuge	RWD Life Science	M1416R
Flow cytometer	Beckman Coulter	CytoFLEX LX (B90883)

### Experimental model and study participant details

#### Institutional permissions

Pediatric peripheral blood was collected under the approved ethical protocol (Ethical Approval No.2024-174) issued by the Ethics Committee of the Children’s Hospital of Chongqing Medical University, Chongqing, China. Informed consent was obtained from all guardians of the participating children.

### Method details

Step-by-step method details can be found in Document S1.

## Supplemental information

Document S1. Materials and equipment, step-by-step method details, troubleshooting, and Figures S1 and S2

Table S1. Granulocyte purity after isolation and purification from individual pediatric peripheral blood samples using the conventional method, related to Step 13

Table S2. Granulocyte yield and purity after isolation and purification from individual pediatric peripheral blood samples using the optimized method, related to Step 13

Table S3. Neutrophil purity after isolation and purification from individual pediatric peripheral blood samples using the optimized method, related to Step 25
