# Supplementary material for: Optimized protocol for isolation of neutrophils from small-volume human blood samples via Percoll density gradient centrifugation
Source: STAR Protoc. 2026 Oct 28;7(4):104914. doi: 10.1016/j.xpro.2026.104914 (PMC13735240; doi:10.1016/j.xpro.2026.104914)
Supplement: Supplementary file 1 [file mmc1.pdf]

Materials and Equipment

Reagent	Final concentration	Amount
Sodium Chloride	1.5 M	4.383 g
Ultra-pure H_2O	N/A	50 mL
**Total**	**N/A**	**50 mL**

Store at 4°C, use within 6 months.

Isotonic Percoll (1× Percoll) solution

Reagent	Final concentration	Amount
Pure Percoll	90%	1.26 mL per sample
1.5 M NaCl solution	10%	0.14 mL per sample
**Total**	**N/A**	**1.4 mL per sample**

The Isotonic Percoll (1× Percoll) solution was prepared fresh before use.

62% Percoll gradient solution

Reagent	Final concentration	Amount
1× Percoll solution	62%	0.62 mL per sample
Saline	38%	0.38 mL per sample
**Total**	**N/A**	**1 mL per sample**

The 62% Percoll gradient solution was prepared fresh before use. Percoll solutions can be stored for up to 6 months under 4 ℃[2].

75% Percoll gradient solution

Reagent	Final concentration	Amount
1× Percoll solution	75%	0.75 mL per sample
Saline	25%	0.25 mL per sample
**Total**	**N/A**	**1 mL per sample**

The 75% Percoll gradient solution was prepared fresh before use. Percoll solutions can be stored for up to 6 months under 4 ℃[2].

PBS (1×) supplemented with 2% FBS

Reagent	Final concentration	Amount
PBS (Tablets)	N/A	10 tablets
FBS	2%	20 mL
Ultra-pure H_2O	N/A	980 mL
**Total**	**N/A**	**1 L**

Store at 4°C, use within 6 months.

PBS (1×)

Reagent	Final concentration	Amount
PBS (Tablets)	N/A	10 tablets
Ultra-pure H_2O	N/A	1 L
**Total**	**N/A**	**1 L**

Store at 4°C, use within 6 months.

Step-by-step method details

<u>*Percoll density separation*</u>

Timing: [~1.5 h]

This section describes the isolation of neutrophils from human peripheral blood via Percoll double-gradient centrifugation, using an initial volume of 1 mL of blood. Blood samples were collected in EDTA-coated blood collection tubes, which were kept at 20–25 °C throughout the period from collection to processing, and the volume may be adjusted within the range of 0.6–2 mL according to sample availability.

NOTE: All reagents were equilibrated to 20–25 °C prior to use, and all experimental procedures were conducted in a biosafety cabinet. Centrifuge acceleration/deceleration: 9/9 unless otherwise indicated.

1. Add 1 mL 75% Percoll into a FACS tube using micropipette.

2. Carefully overlay 1 mL 62% Percoll on top of 75% Percoll using micropipette (P200 recommended) (Figure 2A).

CRITICAL: Add 62% Percoll as a fine stream slowly along one side of the FACS tube's inner wall to avoid the formation of smoke-like, irreversible mixing.

3. Use saline to dilute 1 mL fresh blood to 2 mL in 2 mL microtube.

NOTE: For initial blood volumes within this range, we supplement the sample with saline to a final volume of 2 mL following the unified protocol standards. For initial volumes greater than 1.5 mL, an optional pretreatment step is applicable: samples are centrifuged at 500 × g for 10 min at 20 ℃ to remove the supernatant plasma, and the remaining sample is then supplemented with saline to 2 mL for subsequent isolation.

4. Carefully overlay 2 mL of diluted blood on top of the 62% Percoll using micropipette (P200 recommended), thus bringing to a total volume of 4 mL in FACS tube (Figure 2A).

CRITICAL: Place a single drop of blood on the inner wall of the FACS tube. Gently touch the blood drop with a pipette tip and guide it along the tube wall to the surface of the 62% Percoll. Once the blood has spread naturally to cover the liquid surface, slowly add the remaining blood along the original sliding track of the initial blood drop to complete sample loading. In addition, two underlay loading modes are available as alternative loading schemes and can yield qualified neutrophil separation after experimental validation. The first loads blood prior to underlaying two Percoll solutions sequentially; the second forms pre-assembled Percoll gradient before sample overlay. Both loading schemes yielded high-purity neutrophil preparations in representative experiments (Figure S1).

5. Centrifuge the FACS tubes at 300 × g (300 × g corresponds to 1477 rpm in our centrifuge) for 12 minutes at 20℃, with the centrifuge set to an acceleration rate of 1 and a deceleration rate of 0.

6. Carefully remove FACS tubes following centrifugation so not to disrupt the gradient.

7. Use micropipettes to aspirate and discard the plasma and saline mixture layer, then first collect PBMCs on top of the 62% Percoll layers and transfer the cells to a new 15 mL conical tube.

8. Use micropipettes to aspirate and discard the 62% layer, then collect neutrophils (positioned between the 75% and 62% Percoll layers) and transfer the cells to a new 15 mL conical tube.
NOTE: Starting from this step, isolated neutrophil samples are subjected to all subsequent downstream manipulations described below.

9. Add PBS supplemented with 2% FBS (or RPMI 1640 medium) to each tube to a final volume of 5 mL. Wash the neutrophils via centrifugation at 400 × g for 10 minutes at 20–25 °C.

10. Remove the supernatant completely and resuspend the cell pellet in 400 μL PBS supplemented with 2% FBS (or RPMI 1640 medium).

11. Transfer the resuspended cells to a new 1.5 mL microtube, then add 20–30 μL of EasySep™ RBC Depletion Reagents and place the tube on a magnetic rack (Tiangen, OSE-MF-01, or, EasySep™ Cell Separation Magnets, 18000) for 5 minutes (Figure S2).

CRITICAL: Erythrocyte depletion constitutes an indispensable step in this protocol. Nevertheless, its single-well configuration restricts simultaneous processing to a single sample, leading to extended overall processing time for multiple samples.

12. Use 200 μL pipette to aspirate supernatant into a new 1.5 mL microtube and then centrifuge at 100 × g for 10 minutes at 4℃.

13. Carefully remove the supernatant and resuspend the cell pellet with appropriate amount of PBS supplemented with 2% FBS (or RPMI 1640 medium) based on the size of pellet.

14. Mix 10 µL of cell suspension and 10 µL of Trypan Blue staining solution and add the mixture to a cell count plate to obtain the cell concentration and viability (Pause point).

Neutrophil staining and acquisition on the flow cytometry

Timing: [~1 h]

This section describes the staining of neutrophils with anti-human CD45 (APC-Cy7), 7-AAD and anti-human CD66b (FITC) to test the purity of isolated neutrophils, using flow cytometer. Any flow cytometer with the correct number of channels/colors and analysis software can be used to perform the steps. Compensation was performed prior to sample acquisition to correct spectral overlap between fluorochromes, including FITC, APC-Cy7, and 7-AAD. All flow cytometry reagents were used at 5 µL per test.

15. Prepare three single-stained compensation control tubes and one unstained control tube using 3×10^5 neutrophils in 100 µL with experimental samples.

a. Unstained control tube: acquire unstained cells to establish the baseline autofluorescence and negative gate for all channels.

b. FITC single-stained tube (anti-human CD66b-FITC): acquire single-positive FITC events, and adjust compensation to eliminate FITC spillover into other channels.

c. APC-Cy7 single-stained tube (anti-human CD45-APC-Cy7): acquire single-positive APC-Cy7 events, and calibrate compensation to correct APC-Cy7 spectral overlap.

d. 7-AAD single-stained tube: acquire 7-AAD-positive events, and perform compensation calibration for the 7-AAD channel to exclude fluorescent crosstalk from FITC and APC-Cy7.

16. Confirm no significant fluorescence spillover across all detection channels after compensation adjustment and record the final compensation parameters for consistent acquisition of all experimental samples.

17. Prepare 5×10^5 neutrophils in 100 µL PBS supplemented with 2% FBS per 1.5 mL microtube for unstained control and staining groups.

NOTE: The concentration of neutrophils could be adjusted via centrifugation and resuspension once the initial concentration is lower than 5×10^6/mL.

18. Perform staining as follows:

a. Stain the cells with 5 µL FITC-conjugated anti-human CD66b antibody.

b. For the isotype control group, add 5 µL FITC-conjugated mouse IgM (κ isotype control) to keep identical total volume across all samples.

NOTE: All test and isotype control samples are co-stained with 5 µL APC-CY7-CD45 and 5 µL 7-AAD.

19. Incubate the mixture on ice for 30 min in the dark to avoid light exposure.

20. Add 500 µL PBS into microtube and centrifuge at 400 × g for 5 minutes at 4°C.

21. Discard supernatant and resuspend cells in 500 µL PBS.

22. Centrifuge at 400 × g for 5 minutes at 4°C.

23. Discard supernatant and resuspend cells in 300 µL PBS, then transfer cells to the FACS tube.

24. Set appropriate voltages for channel used for APC-CY7 (R763), 7-AAD (B690) and FITC (B525) with prepared unstained and stained samples (Step 23).

25. Unstained and stained samples (Step 23) are now ready for acquisition.

NOTE: For best results, we recommended acquiring the samples within an hour of staining to avoid confounding results due to cell activation/damage or death.

Troubleshooting

Problem 1

Variable yield of neutrophils after centrifugation (steps 1–14).

Potential solution

This protocol performs optimally when blood is used within 8 hours of collection. All reagents should be equilibrated to 20–25 °C prior to use. While this occurs infrequently, donor-to-donor variability may result in relatively variable neutrophil yields. Given possibility of partial neutrophil loss resulting from elevated neutrophil fractions in KD specimens, centrifugation time can be adjusted accordingly between 12 and 15 min according to CBC neutrophil indices and clinical diagnosis. Samples with abnormally high neutrophil counts linked to immune disorders may require modest time extension within this mentioned range to promote adequate granulocyte sedimentation into the target layer and reduce occasional visible residual red blood cells adhering beneath the granulocyte band. Nevertheless, a 12-min spin remains sufficient for all tested samples to date.

Problem 2

Too much debris in isolated neutrophils (steps 1–14).

Potential solution

Perform the step 12 and 13 again to reduce the debris, though loss of cells is inevitable.

Problem 3

Red blood cells (RBC) contamination

Potential solution

Residual red blood cells will compromise the purity of isolated neutrophils as determined by flow cytometry. Thus, confirm the complete removal of red blood cells by inspecting the color of the cell pellet obtained after Step 12.

Problem 4

Granulocyte layer too thin and difficult to harvest

Potential solution

For excessively thin granulocyte layers hard to retrieve, most of the 62% Percoll solution is first aspirated with a 200 μL pipette tip. A new pipette tip is then used to slowly draw residual 62% Percoll, the indistinct granulocyte interphase and a small portion of underlying 75% Percoll along the inner wall of the FACS tube.

Problem 5

Peripheral blood mononuclear cell (PBMC) contamination

Potential solution

Vibration during solution layering and granulocyte recovery will trigger PBMC infiltration into the granulocyte layer and cause unwanted contamination. It is critical to keep FACS tubes free from shaking throughout the procedure.

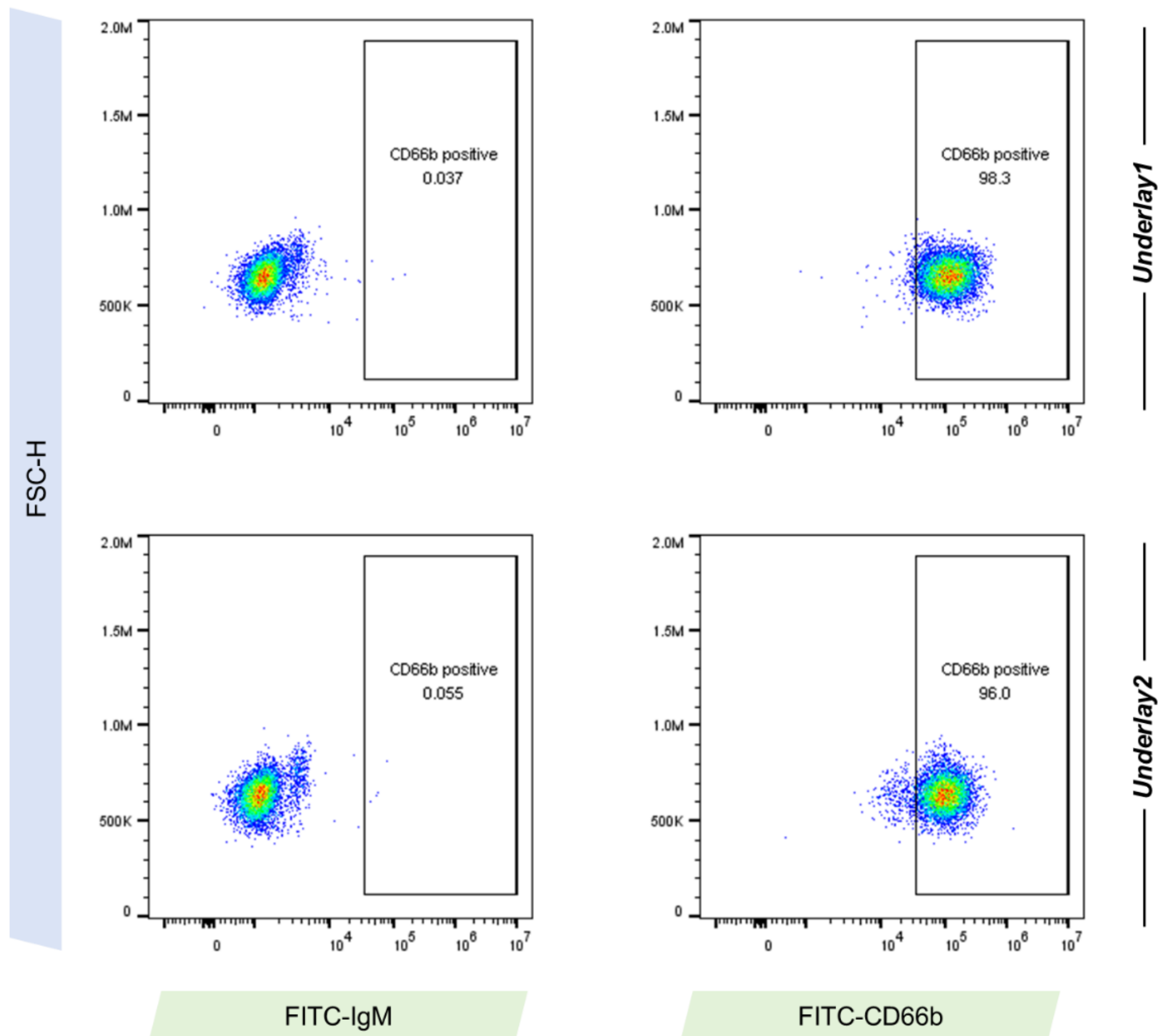

Figure S1: Neutrophil isolation performance of two different underlay loading strategies with Percoll density gradient centrifugation, related to Step 4. Two distinct underlay loading protocols were applied for neutrophil separation using 62% and 75% Percoll density gradient solutions. The first protocol involved initial loading of blood samples followed by sequential underlay of 62% and 75% Percoll solutions beneath the samples. The second protocol was conducted by first loading 62% Percoll solution, followed by underlaying 75% Percoll beneath the 62% layer, and finally overlaying peripheral blood samples on top of the 62% Percoll. Representative flow plots demonstrated that both underlay approaches successfully achieved effective isolation of high-purity neutrophils from human peripheral blood samples.

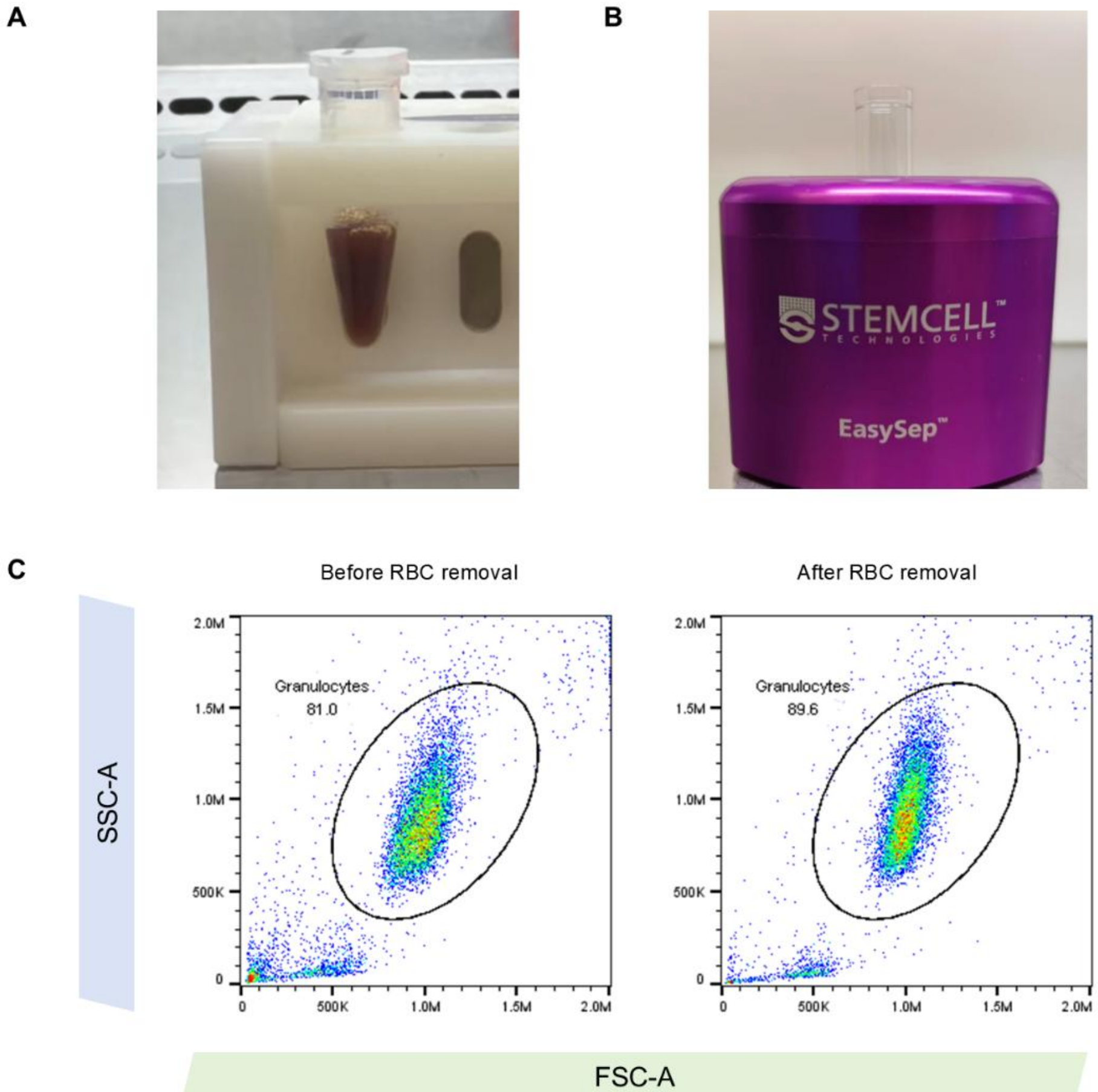

Figure S2: Magnetic bead-mediated red blood cell depletion was performed to remove the erythrocytes from peripheral blood samples post neutrophil isolation, related to Step 11. (A and B) The experiments were separately conducted using two commercial magnetic stands, including the TIANGEN magnetic stand and the Stem Cell magnetic stand, with consistent sample processing. (C) Comparative flow plots illustrating cell composition changes before and after erythrocyte depletion, demonstrating the purification effect of red blood cell removal on isolated granulocyte samples.
